# Using Bayes’ Rule for Analysis of Microfluidic Particle and Cluster Sorting

**DOI:** 10.3390/mi17040396

**Published:** 2026-03-25

**Authors:** Elham Akbari, Esra Yilmaz, Christelle N. Prinz, Jason P. Beech, Jonas O. Tegenfeldt

**Affiliations:** 1 Department of Physics, Division of Solid State Physics, Lund University, P.O. Box 118, SE-221 00 Lund, Sweden; elham.akbari@fysik.lu.se (E.A.); esra.yilmaz@fysik.lu.se (E.Y.); christelle.prinz@fysik.lu.se (C.N.P.); jason.beech@fysik.lu.se (J.P.B.); 2 NanoLund, Lund University, P.O. Box 118, SE-221 00 Lund, Sweden; 3 SciLife Lab, Lund University, P.O. Box 118, SE-221 00 Lund, Sweden

**Keywords:** deterministic lateral displacement, microfluidics, sorting, Bayes’ rule

## Abstract

Deterministic lateral displacement (DLD) and related microfluidic sorting devices are typically evaluated based on the size distributions of particles collected at each outlet, even though the more relevant measure of performance is the probability that a particle of a given size ends up in a specific outlet. Here, we use Bayes’ rule to infer these size-dependent routing probabilities from experimentally accessible measurements of outlet size distributions, inlet size distributions, and outlet subpopulations. Using a DLD array designed to separate microspheres and microsphere clusters, we determine the probabilities that particles of different sizes are directed to each outlet and define a probabilistic critical size ($D_{C}$) at which particles are equally likely to follow a zigzag and a displacement trajectory. Based on this, we calculate key performance metrics, purity, and yield. Our results demonstrate high-quality separations and show that routing probabilities provide a general and robust framework for benchmarking microfluidic sorting devices beyond traditional outlet-based analyses.

## 1. Introduction

A common theme in microfluidic sorting devices is that the unsorted sample is introduced in one end of a long channel and exits into several outlet reservoirs at the end of the channel. Examples include inertial focusing [1], viscoelastic focusing [2], pinched flow fractionation [3], and deterministic lateral displacement (DLD) [4]. To assess the performance of these types of devices, different approaches are employed. The performance of a sorting device is multifaceted and must be described using a broad range of performance indicators depending on the application at hand [5]. Throughput is essential for preparative applications. It can be related to inlet and outlets volumes, the number of cells processed, the number of cells produced, and time to answer. Purity is essential for some preparative applications such a cell therapy where no contamination is allowed [6]. The definition of purity varies with whatever goals are on the table. To start with, one can consider a device-centric definition, such that for a device with cut-offs that define a subpopulation, purity is simply the number of cells within the cut-off range divided by the number of all cells. As an alternative, one can consider a goal-oriented definition that is defined on the needs of the application at hand. In this case, purity is simply the number of *desired* cells divided by the total number of cells. Capture rate (or yield), defined by the fraction of the desired cells that are collected, is important for rare cells as is enrichment ratio [7]. Resolution can be quantified by the overlap of the relevant probability distributions.

In the most common situations, the compositions in the outlets are known, i.e., the probability distribution of sizes within an outlet, P(size|outlet). However, to evaluate the separation performance, what is really needed is the probability P(outlet|size) of a particle of a given size to end up in a specific outlet, i.e., among all particles of a given size, what fraction goes to respective outlet. We call these probabilities the routing probabilities.

In our study, we use data from sorting devices based on DLD. It is a method that is based on the flow of particles through a regular array of posts. The particles ideally follow one out of two distinct trajectories. Particles with a diameter less than a critical diameter, ($D<D_{C}$), follow the flow through the device, in the zigzag mode, while particles with diameters greater than the critical diameter, ($D>D_{C}$), follow a direction determined by the orientation of the array, in the displacement model [4].

The array, in turn, can be defined in one out of two major ways. From a design perspective, it is more straightforward to use a square array that is rotated slightly within the flow channel. For simulations, and for devices based on multiple sequential DLD arrays with different $D_{C}$, the rhombic array is more convenient [8]. To estimate the critical size, a simple expression can be used, that is an approximation of an empirical expression reported by Davis [9]. The approximate expression is $D_{C}=G\sqrt{\left(2/N\right)}$, where *G* is the gap between the posts and *N* is the periodicity of the DLD array. The periodicity is related to the orientation angle of the array as follows: tanθ=1/N.

While the simple picture of a DLD device comprises a binary and deterministic sorting mechanism, real devices are more complex, making it difficult to identify a sharp threshold size. This is due to inherent properties of the DLD design in itself as well as due to the complexity of relevant particles. For small particles, diffusion makes the transition between zigzag and displacement mode gradual [4,10,11]. For array periodicities that are not integers, anomalous trajectories can appear [10]. Specifically for rhombic arrays, anisotropic permeability of the array results in a broadening of the transition between zigzag and displacement modes [12,13,14]. This is predicted to happen also for square arrays for high Reynolds numbers, but not for low Reynolds numbers [15]. Finally, boundary effects, such as wall effects, can be significant, especially for narrow devices [16]. These can be compensated for by careful design of the structure of the walls [17].

Many relevant biological samples are not spherical hard particles. Their shape and deformability will affect the way they move through the DLD array in ways that are often difficult to predict using a simple equation [18,19,20,21,22,23,24]. Some shapes leads to chaotic motion, and if particles aggregate, they can change shape dynamically as they move through the device. Particle–particle interactions are another mechanism that is highly relevant for undiluted samples. Separating particles in blood, the effective $D_{C}$ shifts and the dispersion of the particle trajectories increase with increasing hematocrit [25].

Together, all this creates an urgent need for a well-defined scheme of characterizing key performance indicators for different types of samples that are handled in DLD devices.

In our recent work, we demonstrated the sorting of bacterial clusters with a broad size distribution using a high-*N* DLD device [26]. Here, we showed that introducing an intermediate outlet between the displacement and zigzag outlets increases purity by collecting particles that exhibit significant dispersion due to their broad variation in shape and size near the device’s critical diameter. For such complex samples, we showed that the device’s effective critical diameter, $D_{C}$, can be determined using routing probabilities. Building on this approach, we now calculate the routing probabilities of single microspheres and microsphere clusters to evaluate device performance when sorting particles with well-defined and controlled properties.

The challenge of finding the routing probability is that it is difficult to directly measure the conditional probability P(outlet|size). Instead, we can readily measure the size distributions in each outlet, P(size|outlet), along with the size distribution of the unsorted particles, P(size), and the fraction of all particles that end up in each outlet, P(outlet). We then use Bayes’ rule (for an introduction, refer to, e.g., the textbook by Gelman et al. [27]) to calculate P(outlet|size) for a DLD device designed to separate microspheres and microsphere clusters based on size.

We show that using routing probabilities, we can expand the traditional definition of the critical size, $D_{C}$, from a threshold size separating two adjacent binary distributions, to a quantitative border between two overlapping outlet distributions. We thus define the $D_{C}$ as the size for which the routing probabilities are equal: $P\left(outlet1|size=D_{C}\right)=P\left(outlet2|size=D_{C}\right)$. Note that this critical diameter is related to but not identical to the $D_{C}$ typically associated with DLD devices [4,28].

Once we have the routing probabilities, along with the measured size distributions, we calculate the various performance indicators, such as purity and yield. Purity corresponds to the fraction of desired particles to the total number of particles in an outlet. Yield is defined as the fraction of particles in the inlet in the desired size range that are sent to the desired outlet.

## 2. Materials and Methods

In this chapter, we describe the particles employed, the device fabrication process, data acquisition, and data analysis.

### 2.1. Microspheres

Microspheres were purchased from formerly Duke Scientific now Thermo Fisher Scientific Inc., Waltham, MA, USA, and their properties are listed belowin Table 1.

**Figure 1:**
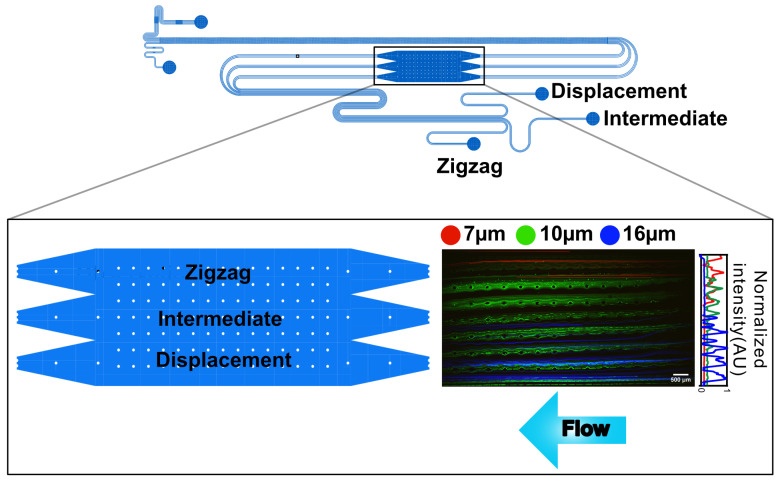
Final lateral position of the different microspheres inside the device’s observation area. Schematic of the device, highlighting the observation area and positions where fluorescent intensities are measured. Close-up view of the observation area (schematics and fluorescence image) with the microspheres moving towards the zigzag outlet, the intermediate outlet, and the displacement outlet. The fluorescence image was created using a maximum-intensity projection and normalized signal intensity (2× magnification). The data are overlays from separate experiments, false-colored to enable diameter distinction (red: 7 μm; green: 10 μm; blue: 16 μm).

Microspheres were diluted by a factor of 100× in a Pluronic^®^ F–127 (MW 12,500 Da, Sigma (Merck KGaA, Darmstadt, Germany)) 0.5% *w*/*v* water solution to minimize aggregation and clogging in the devices and were run in 2 sets of experiments (7 μm and 10 μm microspheres together and 16 μm in another experiment). The 7 μm and 16 μm microspheres share the same excitation wavelength. Therefore, they were tested in separate experiments to avoid mixing in the observation area where the normalized intensity was measured (Figure 1). All experiments were performed 3 times and the data was pooled together.

### 2.2. DLD Device Design

We tested the routing probability analysis on the DLD device used in our earlier study for bacteria cluster sorting [26]. The device features one rotated square array with a high periodicity (N=60). The rotated square array was made of circular pillars of diameter D=50μm, with a gap between the pillars of G=45μm (λ=D+G=95 μm) and a tilt angle with respect to the flow direction θ=0.83°, which gives a critical diameter $D_{C}\approx 8\;$μm, calculated based on Davis’ equation [9].

**Figure 2:**
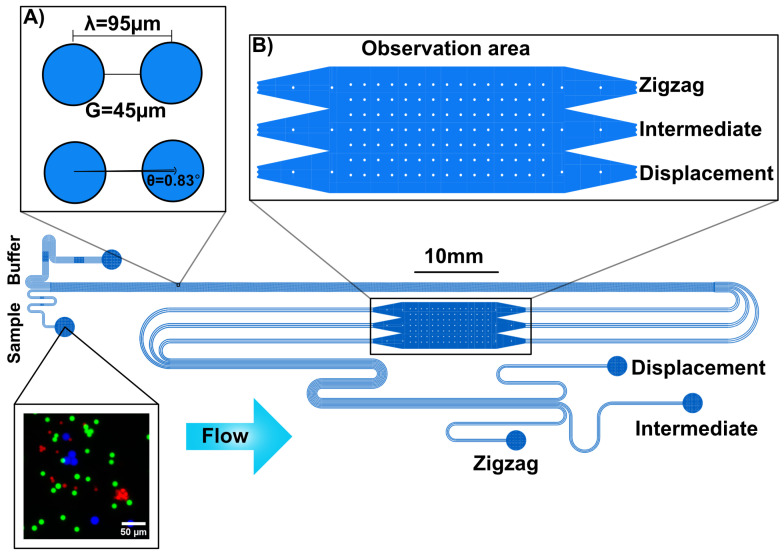
Schematic of the DLD device used in this study along with a false-color image of a typical inlet sample (red: 7 μm; green: 10 μm; blue: 16 μm). (**A**) Key dimensions in the rotated square array device. The device has two inlets (buffer and sample inlet), an observation area, and three outlets (zigzag, intermediate, and displacement). Particles smaller than $D_{C}$ move in zigzag mode and exit the device via the zigzag outlet. Particles larger than $D_{C}$ move in the displacement mode and are collected in the displacement outlet. If there are any other particles that are moving not in displacement nor in zigzag, they are expected to collect in the intermediate outlet. (**B**) The device also contains an observation area, used to observe the lateral particle distributions at the end of the array and before collection in the outlets.

The device has two inlets, three outlets (zigzag, intermediate, and displacement), and an observation area where the flow velocity is 10 times lower than in the rest of the device (Figure 2). The observation area is placed to track the lateral positions of the particles after being sorted by the DLD array and before collection in the outlets. This is used to verify a stable and balanced flow throughout the device during the experiment.

The inlets consist of one sample inlet and one buffer inlet. The buffer inlet consists of seven parallel channels, and the sample inlet consists of three parallel channels, each with a width of 50μm. With equal flow speed between sample and buffer at the entrance of the DLD device, the sample is diluted to 30% of its concentration in the sample inlet.

The buffer and sample inlet channels have equal fluidic resistances per unit width and the same applies to the three outlet channels. However, the outlet channels are longer than the inlet channels, which increases their resistance, which we found helps stabilize the flow. By balancing the resistances at the inlets and outlets, the flow is guided to enter and exit the array in the same direction, remaining parallel to the side walls.

### 2.3. DLD Device Fabrication

A master mold was prepared using 100 μm thick SUEX^®^ dry film resist (K100, DJ Microlaminates, Sudbury, MA, USA), which was laminated onto a 4 inch silicon wafer with a laminator (Catena 35, Acco UK Ltd., Buckinghamshire, UK) at 65 °C. The laminated wafer was then baked on a hotplate (Model 1000-1 Precision Hot Plate, Electronic Micro Systems Ltd., West Midlands, UK) at 85 °C for 5 min to eliminate trapped air and allow the resist to relax. The channel layout was patterned onto the resist using a photomask (Delta Mask B.V., Enschede, The Netherlands). UV exposure was performed at 365 nm in a contact mask aligner (Karl Suss MJB4 Soft UV, Munich, Germany) for 34 s with a lamp intensity of 30 mW/cm^2^, followed by a post-exposure bake at 85 °C for 5 min. Development of the exposed SU-8 was carried out in mr-DEV 600 (Micro Resist Technology GmbH, Berlin, Germany) for 15 min, followed by an additional 5 min in fresh developer. The mold was then rinsed in flowing IPA and dried under nitrogen to remove any unpolymerized resist.

A final hard bake at 200 °C for 2 h in a convection oven completed the master. To avoid the PDMS sticking during replica molding, the surface was coated with a 1 nm aluminum oxide layer and subsequently functionalized with a perfluorodecyltrichlorosilane (FDTS) monolayer using a Fiji Plasma-Enhanced ALD system (Veeco Instruments Inc., Plainview, NY, USA).

The PDMS devices were fabricated from this master by standard replica molding following the method of Xia et al. [28]. The PDMS (Sylgard 184, Dow Corning, Midland, MI, USA) was cast, cured, and then peeled from the master. Inlet and outlet holes were punched prior to bonding. Oxygen–plasma activation (Zepto, Diener electronic GmbH & Co. KG, Ebhausen, Germany) was applied for 15 s to the PDMS and 50 s + 15 s to the glass substrates, after which the two surfaces were brought into contact to seal the channels. Immediately after bonding, the channels were filled with poly(acrylamide)-*g*-(PMOXA™, 1,6-hexanediamine, 3-aminopropyldimethylsilanol) (PAcrAm_50_-*g*-(PMOXA™, NH_2_, Si)) (SuSoS AG, Dübendorf, Switzerland) for passivation of the channel surfaces to avoid the sticking of the microspheres.

### 2.4. Fluidics and Sample Handling

Flow through the device was established by leaving the outlets at atmospheric pressure while supplying nitrogen overpressure to the inlets. These pressures were finely tuned to produce a uniform, stable flow along the pillar array. The sample and buffer were pressurized in two 15 mL tubes using an MFCS-4C controller (Fluigent, Paris, France), set to 140 mbar and 150 mbar, respectively. An overview of the DLD sorting setup is shown in Figure S1 in the SI. Under these conditions, the flow rate was 30±2μL/min, giving an average shear rate of 210 s^−1^ and, assuming water-like viscosity, a shear stress of approximately 0.2 Pa. This falls within commonly used ranges for microfluidic perfusion systems [29], and for example, biofilm experiments, and physiological airway shear.

The fluids were delivered to the device through capillaries with a 768 μm inner diameter. Before introducing samples, the channels were conditioned by flushing with running buffer (0.5% *w*/*v* Pluronic^®^  F–127) for 10 min.

### 2.5. Imaging and Analysis

All experiments were performed under observation using an inverted epifluorescence microscope (Nikon Eclipse TE2000-U, Nikon Corporation, Tokyo, Japan) in transmission mode with a scientific CMOS camera (Andor NEO sCMOS, Andor Technology, Belfast, Northern Ireland), model number DC-152Q-FI, 16 bit, pixel size 6.5 μm, sensor size 16.6 mm × 14.0 mm, 2560 × 2160 pixels, and 100fps rolling shutter. The following objectives were used: 2× Nikon Plan UW, NA 0.06; 4× Nikon Plan Apo λ, NA 0.2; 10× Nikon Plan Apo λ, NA 0.45; 20× Nikon Plan Fluor, NA 0.45. Imaging was performed using FITC and Cy5 filter cubes and an LED light source (Sola light engine, Lumencore, Beaverton, OR, USA).

For the separation experiments, batches of microscopic images from the inlet and outlet reservoirs were processed using a custom automated image-analysis workflow. In addition to basic segmentation and object characterization, the workflow incorporated normalization by the fluorescent lamp profile, background subtraction, compensation for spectral crosstalk between fluorescence channels, and identification of overlapping objects.

The pipeline was implemented in Python ver. 3.8 (Spyder/Anaconda 4) using packages including numpy, matplotlib, skimage (particularly skimage.measure with RegionProps), cv2, scipy, and pandas, and Meta Segment Anything Model 2 (SAM 2) [30].

### 2.6. Microsphere Characterization in the Various Reservoirs

All separation experiments were performed 3 times, and the data were pooled. For microsphere and microsphere cluster characterization, the 7 μm and 10 μm microspheres were imaged with the 20× objective, while the 16 μm microspheres were imaged with the 10× objective.

The microspheres were imaged directly in the three outlets, each containing 300 μL of microsphere suspension. For characterization of the sample inlet, 10 μL of the solution was pipetted onto a glass slide, covered with a coverslip, and imaged. Taking into account dilution by the buffer, which reduced the sample concentration to 30% of its initial value at the device entrance, capture rates were evaluated for each nominal diameter population as the sum of the concentrations in the three outlets divided by 30% of the concentration in the sample inlet.

For the 7 μm microspheres, 1680 particles were segmented and measured in the zigzag outlet and 1420 in the intermediate outlet. For the 10 μm microspheres, 1005 particles were measured in the zigzag outlet, 2500 in the intermediate outlet, and 1660 in the displacement outlet. For the 16 μm microspheres, 1140 particles were measured in the intermediate outlet and 3900 in the displacement outlet (see Tables S2 and S3 in the SI).

The uncertainty in particle counting was estimated to be 10–20%, primarily due to sedimentation in the reservoirs and tubing. Additional losses were attributed to clogging inside the device.

## 3. Results and Discussion

### 3.1. Sorting Result

The ability of the device to separate microspheres was evaluated for each microsphere diameter. The lateral positions of the particles were measured as they flowed through the observation area, and the microspheres were counted in the three outlet reservoirs. Figure 1 shows the superposed, time-averaged, false-colored fluorescence micrographs of the microspheres in the observation area. The background signal was subtracted, and each color corresponds to a specific microsphere diameter. Note that the data include both singlets and clusters of microspheres formed during long-term storage in the vials.

The distribution of microspheres within the width of the observation area shows a separation of the particles by the DLD arrays. The majority of the 7 μm microspheres exit through the zigzag, while almost all 16 μm microspheres exit through the displacement region. A total of 10 μm microspheres with a size distribution close to the device’s $D_{C}$ exit through the intermediate and zigzag paths.

**Figure 3:**
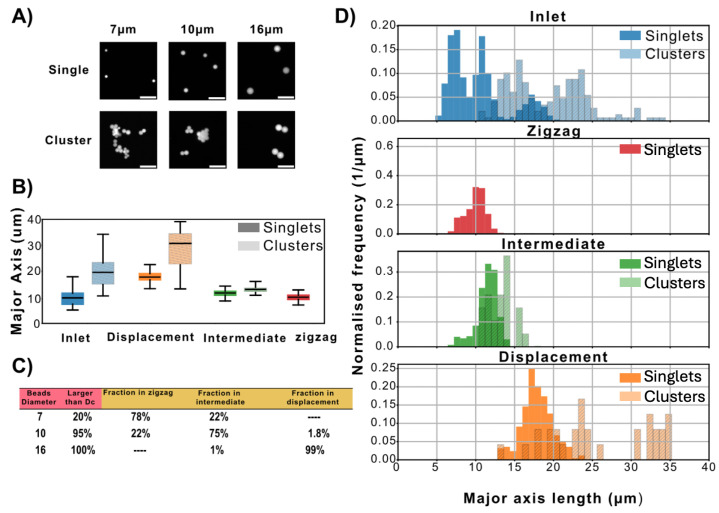
Inlet composition and outlet content after separation of microspheres in the device. (**A**) Representative fluorescence images of single microspheres and clusters of nominal diameter 7, 10, and 16 μm. Scale bars: 30 μm. (**B**) Boxplot of the major axis length of the microspheres and microsphere clusters present in each reservoir. The box shows the 25th, 50th, and 75th percentile; the whiskers show the upper and lower limits. Note that for any spherical particle, the major axis corresponds to the diameter. The two grayscale levels in the legend refer to the use of saturated colors for singlets and less saturated colors for clusters. Color scheme for the different particle sizes and for singlets and clusters is the same as for panel (**D**). (**C**) Fraction of sorted microspheres in each outlet for each nominal diameter population. The second column shows the percentage of particles larger than the nominal $D_{C}\approx 8\;$μm in the inlet before separation, as measured using fluorescence microscopy. Columns 3–5 show the total fraction of microspheres and clusters of each nominal diameter found in each the outlets. (**D**) Histogram of size values of the microspheres in the inlet and the three outlets. The histogram shows the normalized counts.

After separation, for each nominal diameter, image analysis was performed to measure the size distributions of particles present in both the unsorted inlet population and the separated subpopulations collected in the three device outlets (Figure 3). Details on image analysis can be found in Section S2 of the SI. For single microspheres, the major axis corresponds to the microsphere diameter.

Based on the data presented in the boxplot and histogram plots in Figure 3B,C, the zigzag outlet contained only single 7 μm and 10 μm microspheres. The intermediate outlet contained single 7 μm and 10 μm microspheres, as well as small clusters (primarily pairs) of 7 μm microspheres. The displacement outlet contained both single particles of 16 μm microspheres and clusters of 7 μm and 10 μm.

The capture rate of the device was estimated for all three microsphere populations. We quantified the fraction of each microsphere type collected in each outlet, and we found that most microspheres entering the device were recovered in the outlets (Table S3 in the SI). The 7 μm and 16 μm microspheres were separated into two subpopulations, above and below the nominal critical size $D_{C}$, whereas the 10 μm microspheres were found in the zigzag and intermediate outlets. This indicates that the actual critical size of the device lies within the size distribution of the 10 μm microspheres. These results demonstrate the ability of the device to separate microspheres and microsphere clusters.

### 3.2. Statistical Analysis

To further characterize the sorting behavior of the DLD device, we used Bayes’ rule to estimate the routing probabilities of particles (singles and clusters) passing through the device. Because this method is applicable to other microfluidic sorting devices (not only DLD), we adopted a general terminology for the contents in the reservoirs: Inlet (corresponding to the unsorted sample), Small (corresponding to the DLD zigzag outlet), Medium (corresponding to the DLD intermediate outlet), and Large (corresponding to the DLD outlet). We define the routing probabilities as the probabilities $P(k|d_{i})$ that a particle of size $d_{i}$ from the inlet is routed to outlet *k*. Here, and in the following, we define k∈{S,M,L} (Small, Medium, Large). For the size $d_{i}$, we used the major axis length, which, in the case of a perfect circle, corresponds to the diameter. Our starting point is the observed probability mass distributions of the unsorted particle sizes in the sample Inlet (*I*) and the sorted particles in the three outlets (S,M,L). We used the convention of conditional probabilities to refer to subsets belonging to a total set of data points. The bin width is equal for all the discrete probability distributions and optimized based on the inlet data using the Freedman–Diaconis rule [31]. Definitions are summarized in Table S4 and illustrated in Figure S4 in the SI.

We derive the routing probabilities for different particle sizes step by step to make it clear where assumptions and simplifications are introduced.

Step 1: We assume that there is no loss in the device and that all particles move from the inlet to the outlets. The routing probability $P(k|d_{i})$ is the probability that a particle of size $d_{i}$ finds its way from the inlet to outlet *k*. It is given by the total number of particles $n_{d_{i},k}$ of size $d_{i}$ present in outlet *k*, divided by the total number of particles of size $d_{i}$ across all outlets, $n_{d_{i}}$.

(1) $$P\left(k|d_{i}\right)=\frac{n_{d_{i},k}}{\sum_{j\in \{S,M,L\}}n_{d_{i},j}}$$

Step 2: The total number of particles $n_{d_{i},k}$ of size $d_{i}$ that exist in outlet *k* is given by the total number of particles $n_{k}$ in outlet *k* multiplied by the fraction $P(d_{i}|k)$ of particles of size $d_{i}$ among the particles in outlet *k*.

(2) $$P\left(k|d_{i}\right)=\frac{n_{k}\;P\left(d_{i}|k\right)}{\sum_{j\in \{S,M,L\}}n_{j}\;P\left(d_{i}|j\right)}$$

**Figure 4:**
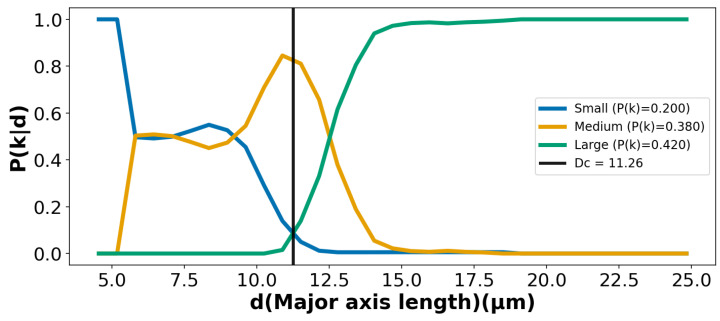
Routing probabilities $P(S|d_{i})$, $P(M|d_{i})$, and $P(L|d_{i})$ to the three outlets for microspheres and clusters, calculated using the values of P(k) given in Table S3 in the SI. The crossover for the Small and Large subpopulations gives $D_{C}$, with a negligible overlap between the two subpopulations.

Step 3: The total number of particles $n_{k}$ that exist in outlet *k* is given by the total number of particles in the device (initially added to the inlet) *N* multiplied by the fraction P(k) of all particles that end up in outlet *k*. It is clear that *N* (total number of particles) is a common factor that is canceled, resulting in the expression that describes what the code evaluates (see Section S2 of the SI for details). The resulting routing probabilities for the DLD device are plotted in Figure 4.

(3) $$P\left(k|d_{i}\right)=\frac{N\;P\left(k\right)\;P\left(d_{i}|k\right)}{\sum_{j\in \{S,M,L\}}N\;P\left(j\right)\;P\left(d_{i}|j\right)}=\frac{P\left(k\right)\;P\left(d_{i}|k\right)}{\sum_{j\in \{S,M,L\}}P\left(j\right)\;P\left(d_{i}|j\right)}$$

Using the measured size distributions in the outlets, $P(d_{i}|k)$, and the fractions of all particles that end up in each outlet, P(k), we can now calculate an effective critical diameter, $D_{C}$, defined by the particle size (major axis length) at which the probability of ending up in the Small subpopulation (zigzag outlet) equals the probability of ending up in the Large subpopulation (displacement outlet).

(4) $${\left.P\left(L|d_{i}\right)=P\left(S|d_{i}\right)\right|}_{d_{i}=D_{C}}$$

For the microspheres and clusters, we obtain $D_{C}\approx 11.3\;\mu$m. This is close to the result based on the single microspheres only, as described in the SI (see Figure S3 and Table S1). For both cases, the Medium particles collected in the intermediate outlet can be regarded as a trade-off, representing a loss that enables higher purity in the Small and Large subpopulations.

We then used the measured size distributions of particles in each outlet together with the calculated routing probabilities to determine the purity and yield of the sorting process.

We define the purity of a subpopulation in a reservoir as the number of particles in the desired size range divided by the total number of particles in that reservoir. The purity of the Small outlet (zigzag) subpopulation, Purity(S), is defined as the fraction of particles in that outlet having a diameter smaller than the critical diameter $D_{C}$. This is obtained by summing the elements of the size probability distribution in the outlet, $P(d_{i}|S)$, from the minimum size (corresponding to i=1) up to $D_{C}$:

(5) $$\mathrm{Purity}\left(S\right)=P\left(d<D_{C}|S\right).$$

Similarly, the purity of the Large outlet (displacement) subpopulation is defined as

(6) $$\mathrm{Purity}\left(L\right)=P\left(d>D_{C}|L\right).$$

The yield of a subpopulation in an outlet reservoir is defined as the fraction of particles in the inlet, within the desired size range, that are routed to the desired outlet. The yield of the Small outlet (zigzag) subpopulation, Yield(S), is defined as the fraction of inlet particles with diameter smaller than the critical diameter $D_{C}$ that are routed to the Small outlet. This is obtained by summing the routing probability distribution $P(S|d_{i})$ from the minimum size up to $D_{C}$:

(7) $$\mathrm{Yield}\left(S\right)=P\left(S|d<D_{C}\right).$$

Similarly, the yield of the Large outlet (displacement) subpopulation is defined as

(8) $$\mathrm{Yield}\left(L\right)=P\left(L|d>D_{C}\right).$$

Due to the difficulty of precisely estimating the fraction of the sample entering the device, we report the yield as a nominal yield, assuming that the total number of particles collected in all outlets equals the total number entering from the inlet. This assumption is supported by the high correspondence between the inlet and total outlet concentrations reported in Table S3 in the SI. However, the reported yield does not provide precise information regarding any selective losses between the inlet and outlet reservoirs.

From the measured size distributions of the microspheres and clusters in the reservoirs (Figure 3) and the routing probabilities (Figure 4), we calculated a purity of 98.1% for the Small subpopulation and 100% for the Large subpopulation, with corresponding yields of 49.6% and 85.2%, respectively. For comparison, we repeated the calculations based on a selection of only the single microspheres. The purities were comparable but yields were found to be greater, possibly due to the more deterministic trajectories of the spherical particles (see Figure S3 and Table S1 in the SI). These values indicate adequate sorting performance. The particles ending up in the intermediate outlet can be regarded as a necessary trade-off to achieve higher purity in the Small and Large subpopulations.

## 4. Conclusions

In this study, we have demonstrated that routing probabilities provide a rigorous and versatile framework for characterizing the performance of DLD devices. By inferring the probability that a particle of a given size reaches each outlet from experimentally measurable size distributions, we capture the complex effects of diffusion, array geometry, and interactions that challenge idealized deterministic models. This approach allows us to define a probabilistic critical size and to calculate key performance metrics. The results highlight that even in the presence of real-world complexities, DLD devices can achieve high-quality separations. More broadly, routing probabilities offer a generalizable method for benchmarking microfluidic sorting systems and can guide the rational design and optimization of devices for diverse applications, from synthetic particle separation to complex biological samples.

## Figures and Tables

**Table 1:** Microspheres used in the experiments.

Color	Diameter (μm)	Coefficient of Variation (CV%)	Lot Number
Green	7	13%	26,920
Red	10	12%	27,150
Green	16	12%	27,640

## Data Availability

Raw data is deposited at Harvard Dataverse: https://dataverse.harvard.edu/dataverse/Bayes_DLD accessed on 11 March 2026 [32]. Code for segmentation is available at GitHub: https://github.com/Elhamakbr/Elhamakbr/blob/main/SAM_segmentation_and_region_props_measurement accessed on 11 March 2026 [33]. Code for estimation of $D_{C}$ is available at GitHub: https://github.com/Elhamakbr/Elhamakbr/blob/main/minimal_model_with_Dc_estimation_and_yield_and_purity_calculation accessed on 11 March 2026 [34].

## Supplementary Materials

The following supporting information can be downloaded at https://www.mdpi.com/article/10.3390/mi17040396/s1, Figure S1: Overview of the DLD sorting setup. Sample and buffer are introduced through capillary tubes. The separated fractions are collected from outlet tubes glued onto the PDMS at ambient pressure. Figure S2: Overview of the particles analysed in our work. (A) Example of particles used in the study. Image based on an overlay of separate images of each type of microsphere. (B) Circularity values versus major axis length of segmented particles. The highlighted region with high circularity indicates approximately which particles are included for the calculations based on the single particles without the clusters. The exact limits are given in Table S1. Figure S3: As a comparison to Figure 4 in the main text, the routing probabilities are given here for single particles by selecting only those particles with circularity, 0.90<c<1.00. For this calculation we assume all equal P(k)=1/3. Figure S4: Schematic overview of relevant subsets of the data based on a representative distribution. (A) We first consider the entire set of particles in the device. This can also be considered the set of particles that are initially introduced into the inlet reservoir. In this partition, we can obtain the total number of particles, *N*, and the size distribution of all the particles, $P(d_{i})$. (B) The experiment sorts the particles into three outlets. We therefore consider subsets defined by the partitioning (vertically in the figure) of the entire set of particles into subsets k∈{S,M,L}. In these partitions, we obtain the total number of particles in each outlet, $n_{k}$, giving $P\left(k\right)=n_{k}/N$, and the size distribution of the particles in each outlet, $P(d_{i}|k)$. (C) We are interested in the probabilities of each size to go to each one of the respective outlets. Therefore, we partition (horizontally in the figure) the set of all particles into subsets based on size. In these partitions, we obtain the total number of particles for each size, $n_{d_{i}}$, and the probability of a particle of a given size to end up in a given outlet, the routing probabilities, $P\left(k|d_{i}\right)=n_{d_{i},k}/n_{d_{i}}$. Table S1: Parameters values used in the code for calculating the routing probabilities and the key performance indicators for two different subsets of the data. Size refers to major axis length. The experimentally derived values of P(k) are taken from Table S3. Table S2: Particle counts in the outlets. A volume of 300 μL was analysed in each outlet. Table S3: Measured concentrations (particles/mL) of microspheres and clusters with calculated capture rates and fractions of all particles going to each respective outlet, P(k). Capture rate is the ratio of the total outlet concentration and the inlet concentration taking into account the dilution by the buffer inlet. P(k) is estimated based on the outlet concentrations. The concentration derived from the Inlet is ≈4% less than for that from the Outlets. Table S4: Definitions of probabilities used in the statistical analysis.
