# Supplementary material for: Using Bayes’ Rule for Analysis of Microfluidic Particle and Cluster Sorting
Source: Micromachines (Basel). 2026 Mar 25;17(4):396. doi: 10.3390/mi17040396 (PMC13118485; doi:10.3390/mi17040396)
Supplement: Supplementary file 1 [file micromachines-17-00396-s001.zip › micromachines-4181936-supplementary.pdf]

Supporting Information for Using Bayes' Rule for Analysis of Microfluidic Particle and Cluster Sorting

Elham Akbari[†1, 2], Esra Yilmaz[†1, 2], Christelle N. Prinz[1, 2, 3], Jason P. Beech[1, 2], and Jonas O. Tegenfeldt[1, 2, *]

[1]Department of Physics, Division of Solid State Physics, Lund University, Lund, Sweden
[2]NanoLund, Lund University, Lund, Sweden
[3]SciLife Lab, Lund University, Lund, Sweden
[*]Corresponding author: jonas.tegenfeldt@fysik.lu.se , Tel.: +46-222 8063

Contents

1 Experimental setup **2**

2 Image and data analysis **3**

3 Inlet and outlet statistics **6**

4 Probabilities **7**

[†]Equal contributions

1 Experimental setup

Figure S1 shows an overview of the deterministic lateral displacement (DLD) sorting setup used in this study. The sample and buffer were introduced into the microfluidic device through capillary tubes connected to the inlets. Inside the device, particles were separated within the DLD pillar array according to their size as they flow through the channel. The separated fractions exit the device through outlet tubes glued onto the PDMS and were observed at ambient pressure for further analysis.

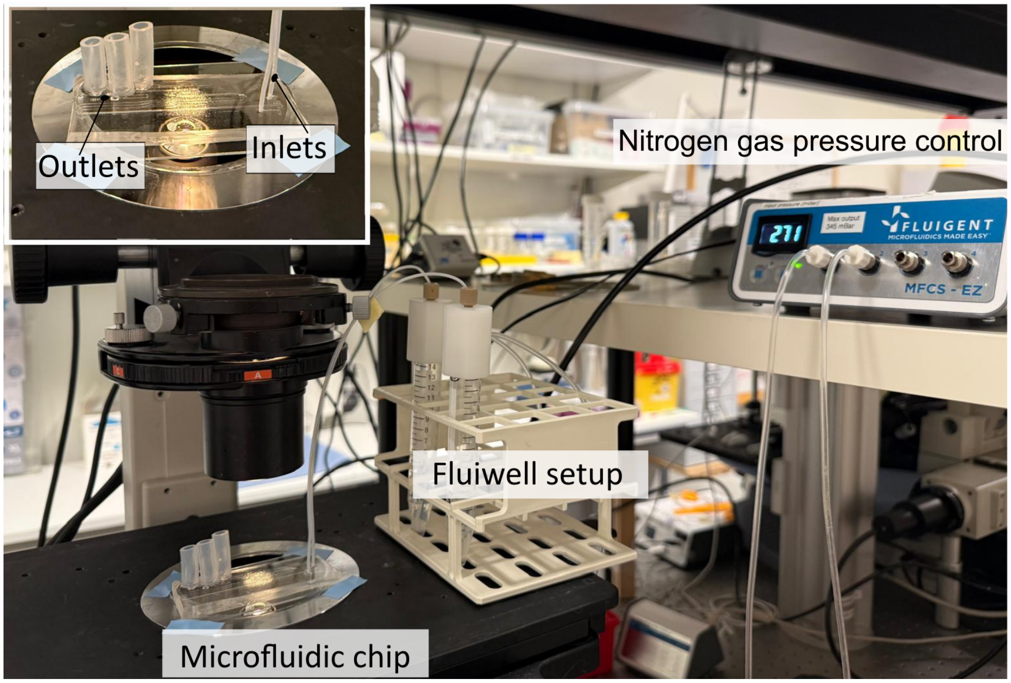

Figure S1: Overview of the DLD sorting setup. Sample and buffer are introduced through capillary tubes. The separated fractions are collected from outlet tubes glued onto the PDMS at ambient pressure.

2 Image and data analysis

After imaging of the particles from the inlet and outlet samples, for image analysis, particles were segmented using the Segment Anything Model 2 (SAM 2), an AI model developed by Meta AI[1]. Raw data and selected processed data has been deposited at Harvard Dataverse[2]. Code was developed for segmentation followed by characterization using Regionprops[3]. Circularity, c, was calculated by comparing the segmented area, A, and the major axis length, d, as given by Regionprops.

$$c = \frac{4A}{\pi d^2} \tag{S1}$$

The routing probabilities shown in the main text, Fig. 4, were computed directly from the measured histograms representing microspheres and clusters of microspheres without any fitting using a minimal model[4]. As a comparison, routing probabilities were also calculated based on a selection of highly circular particles, mostly single microspheres. See Fig. S2. The same code was used for both cases but with different parameter settings as given in Table S1. Here also the resulting performance indicators are listed for the two cases.

Table S1: Parameters values used in the code [4] for calculating the routing probabilities and the key performance indicators for two different subsets of the data. Size refers to major axis length. The experimentally derived values of $P(k)$ are taken from Table S3 .

	Fig. 4	Fig. S3
Parameter	singles & clusters	singles
$P(S)$	0.20	0.33
$P(M)$	0.38	0.33
$P(M)$	0.42	0.33
Size range (μm)	2 .. 25	2 .. 25
Circularity range	0 .. 1.00	0.90 .. 1.00
D_C (μm)	11.26	11.40
Purity(S)	98.1%	98.6%
Purity(L)	100%	100%
Yield(S)	49.6%	64.7%
Yield(L)	85.2%	85.2%

In the following the main steps in the code [4] are laid out. First, the counts, n_k, in each outlet for a given volume were binned and normalized to obtain the conditional size distributions $P(d_i \mid k)$. Here and in the following, k refers to the three outlets, thus $k \in \{S, M, L\}$. Note that all probability vectors represent normalized probability mass distributions, i.e. all vector elements add up to one. See Section 4 and Table S4 for details. For technical reasons in the code, the inlet histogram was converted into a probability density $c_1 P(d_i)$ with equal sized bins. Note that therefore the normalization of the probability *density* must take into account the size of the bins, w_i. c_1 and c_2 are positive constants.

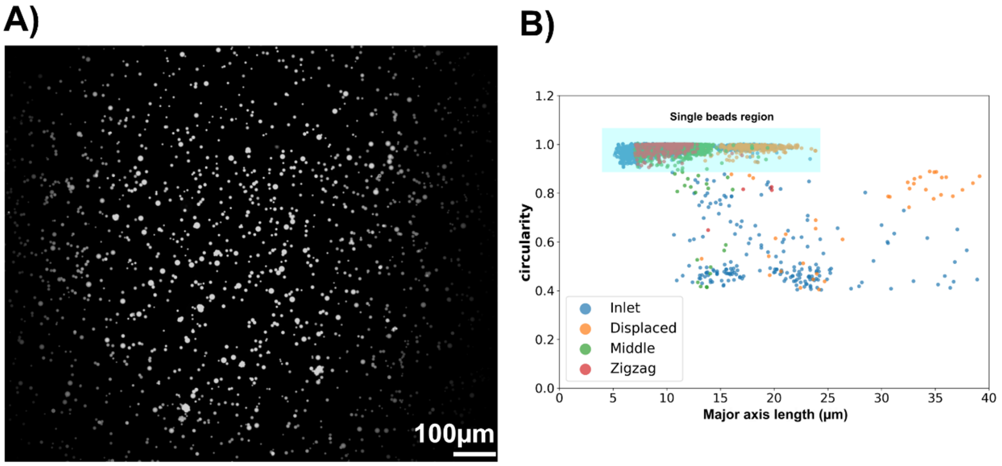

Figure S2: Overview of the particles analysed in our work. (A) Example of particles used in the study. Image based on an overlay of separate images of each type of microsphere. (B) Circularity values versus major axis length of segmented particles. The highlighted region with high circularity indicates approximately which particles are included for the calculations based on the single particles without the clusters. The exact limits are given in Table S1.

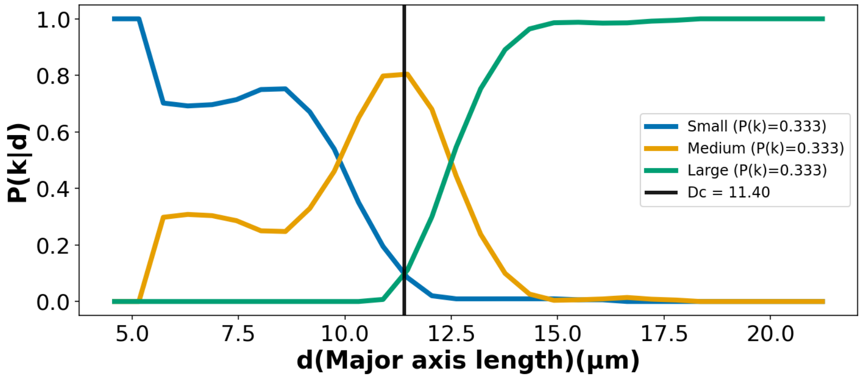

Figure S3: As a comparison to Fig. 4 in the main text, the routing probabilities are given here for single particles by selecting only those particles with circularity, $0.90 < c < 1.00$. For this calculation we assume all equal $P(k) = 1/3$.

$$\sum_i c_1 P(d_i) \times w_i = 1 \tag{S2}$$

Next step is that for each size bin, the ratio $c_1 P(d_i \mid k)/P(d_i)$ was calculated, yielding an un–normalized quantity proportional to $P(k \mid d_i)$.

$$c_2 P(k \mid d_i) = \frac{P(k)P(d_i \mid k)}{c_1 P(d_i)} \tag{S3}$$

These values were then normalized across outlets for each bin to ensure that we have $\sum_k P(k \mid d_i) = 1$.

$$\frac{c_2 P(k \mid d_i)}{\sum_{j\in\{S,M,L\}} c_2 P(j \mid d_i)} = \frac{P(k)P(d_i \mid k)/c_1 P(d_i)}{\sum_{j\in\{S,M,L\}} P(j)P(d_i \mid j)/c_1 P(d_i)} \tag{S4}$$

The proportionality constants and $P(d_i)$ cancel. By definition, $\sum_k P(d_i \mid k) = 1$. The code is thus evaluating Eq. S5 (Eq. 3 in the main text) with all $P(k)$ equal or with values for $P(k)$ from Table. S1.

$$P(k \mid d_i) = \frac{P(k)P(d_i \mid k)}{\sum_{j\in\{S,M,L\}} P(j)P(d_i \mid j)} \tag{S5}$$

If all $P(k)$ equal they cancel in Eq. S5, and we have instead Eq. S6. Note that the summation is over the outlets.

$$P(k \mid d_i) = \frac{P(d_i \mid k)}{\sum_{j\in\{S,M,L\}} P(d_i \mid j)} \tag{S6}$$

As an alternative, one can include the measured inlet distribution, $P(d_i)$, directly in the analysis by noting that in Eq. S5, the denominator equals the normalized size distribution of the entire set of particles, $P(d_i)$.

$$P(k \mid d_i) = \frac{P(k)\,P(d_i \mid k)}{P(d_i)} \tag{S7}$$

Smoothing and monotonicity constraints were applied afterward for numerical stability and visualization. No prior weighting, exposure correction, or iterative optimization was used in this model.

3 Inlet and outlet statistics

Particles were counted in the outlets summarized in Table S2. Based on Poisson statistics, we see that our sampling error for each category is less that 3.2%.

Table S2: Particle counts in the outlets. A volume of 300 μL was analysed in each outlet.

	7 μm	10 μm	16 μm	TOTAL
Counts in Small	1680	1005	–	2685
Counts in Medium	1420	2500	1140	5060
Counts in Large	–	1660	3900	5560
TOTAL	3100	5165	5040	

Particle concentrations are calculated based on the counts in the outlets (Table S2) and based on inlet counts acquired similarly. Note that the contents of the sample inlet is diluted by the buffer from the buffer inlet. The buffer inlet consists of seven parallel channels, and the sample inlet consists of three parallel channels, each channel $50\mu m$ wide. With equal flow speed between sample and buffer at the entrance of the DLD device, the sample is diluted to 30% of its concentration in the sample inlet. The resulting particle concentrations along with calculated capture rates and $P(k)$ are shown in Table S3. Concentrations are calculated taking into account the analyzed volumes.

Table S3: Measured concentrations (particles/mL) of microspheres and clusters with calculated capture rates and fractions of all particles going to each respective outlet, $P(k)$. Capture rate is the ratio of the total outlet concentration and the inlet concentration taking into account the dilution by the buffer inlet. $P(k)$ is estimated based on the outlet concentrations. The concentration derived from the Inlet is $\approx 4\%$ less than for that from the Outlets.

	7 μm	10 μm	16 μm	TOTAL	P(k)
Concentration in Inlet	30000	58000	54000	142000	–
Concentration in Small	5600	3350	–	8950	0.20
Concentration in Medium	4700	8300	3800	16800	0.38
Concentration in Large	–	5530	13000	18530	0.42
TOTAL (Outlet)	10300	17180	16800	44280	1.00
Capture rate	114%	99%	104%	104%	–

4 Probabilities

The probabilities that we work with can be interpreted as histograms. They are vector identities where each element represents the probability for a small range of sizes (bin width). All bins have identical width, and each probability distribution sums to one. In other words, all elements in each vector add up to one. Each vector corresponds to a discrete probability mass distribution. See Table S4 and Fig. S4 for definitions.

Table S4: Definitions of probabilities used in the statistical analysis.

Probability size distribution in Inlet	$P(d_i) = P(d_i \mid I)$
Probability size distribution in outlet Small	$P(d_i \mid S)$
Probability size distribution in outlet Medium	$P(d_i \mid M)$
Probability size distribution in outlet Large	$P(d_i \mid L)$
Fraction of Inlet population found in Small	$P(S)$
Fraction of Inlet population found in Medium	$P(M)$
Fraction of Inlet population found in Large	$P(L)$
Probability of size d_i going to outlet Small	$P(S \mid d_i)$
Probability of size d_i going to outlet Medium	$P(M \mid d_i)$
Probability of size d_i going to outlet Large	$P(L \mid d_i)$

The bin widths, w_{bin}, for all the discrete probability mass distributions are calculated based on the Freedman–Diaconis rule using the interquartile range ($Q75 - Q25$), IQR, and the number of observations in the inlet data, n. The Freedman–Diaconis rule is robust, using IQR makes it insensitive to outliers, and it minimizes the squared difference with the underlying probability distribution[5].

$$w_{bin} = 2 \times \frac{IQR}{\sqrt[3]{n}} \tag{S8}$$

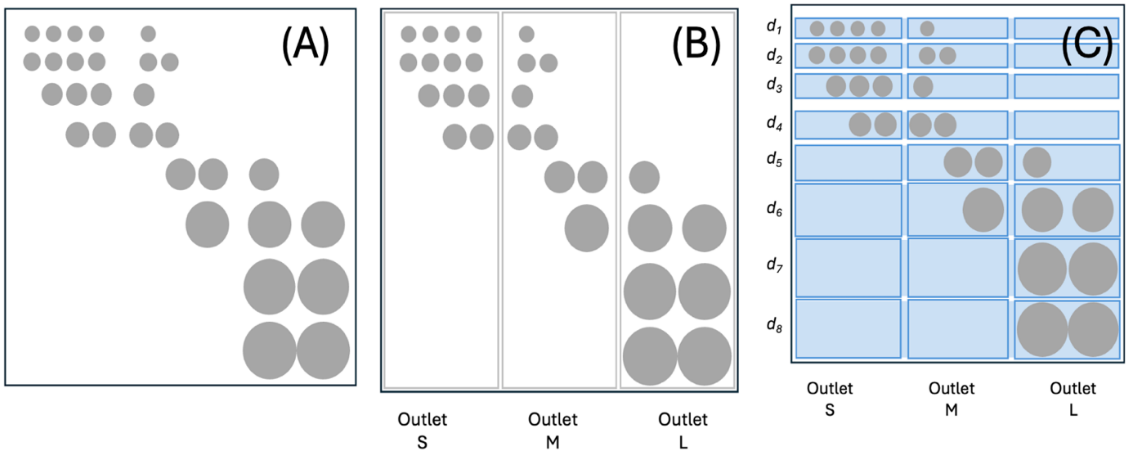

Figure S4: Schematic overview of relevant subsets of the data based on a representative distribution. (A) We first consider the entire set of particles in the device. This can also be considered the set of particles that are initially introduced into the inlet reservoir. In this partition, we can obtain the total number of particles, N, and the size distribution of all the particles, $P(d_i)$. (B) The experiment sorts the particles into three outlets. We therefore consider subsets defined by the partitioning (vertically in the figure) of the entire set of particles into subsets $k \in \{S, M, L\}$. In these partitions, we obtain the total number of particles in each outlet, n_k, giving $P(k) = n_k/N$, and the size distribution of the particles in each outlet, $P(d_i \mid k)$. (C) We are interested in the probabilities of each size to go to each one of the respective outlets. Therefore, we partition (horizontally in the figure) the set of all particles into subsets based on size. In these partitions, we obtain the total number of particles for each size, n_{d_i}, and the probability of a particle of a given size to end up in a given outlet, the routing probabilities, $P(k \mid d_i) = n_{d_i,k}/n_{d_i}$.

References

[1] Alexander Kirillov, Eric Mintun, Nikhila Ravi, Hanzi Mao, Chloe Rolland, Laura Gustafson, Tete Xiao, Spencer Whitehead, Alexander C Berg, Wan-Yen Lo, et al. Segment anything. In *Proceedings of the IEEE/CVF international conference on computer vision*, pages 4015–4026, 2023.

[2] Elham Akbari. Raw data deposited at Harvard Dataverse. `https://dataverse.harvard.edu/dataverse/Bayes_DLD`. Accessed: 2026-03-11.

[3] Elham Akbari. Segmentation using SAM (Meta AI). `https://github.com/Elhamakbr/Elhamakbr/blob/main/SAM_segmentation_and_region_props_measurement`. Accessed: 2026-03-11.

[4] Elham Akbari. D_C−estimation. `https://github.com/Elhamakbr/Elhamakbr/blob/main/minimal_model_with_Dc_estimation_and_yield_and_purity_calculation`. Accessed: 2026-03-11.

[5] David Freedman and Persi Diaconis. On the histogram as a density estimator: L_2 theory. *Zeitschrift für Wahrscheinlichkeitstheorie und Verwandte Gebiete*, 57(4):453–476, 1981.
